# Diverging Trends in Home Respiratory Care in an Aging Society: National Data from Japan

**DOI:** 10.31662/jmaj.2025-0583

**Published:** 2026-06-12

**Authors:** Tomohiro Akaba, Hideki Katsura, Miki Kubo, Etsuko Tagaya

**Affiliations:** 1 Department of Respiratory Medicine, Tokyo Women’s Medical University School of Medicine, Tokyo, Japan; 2 Department of Clinical Epidemiology and Health Economics, School of Public Health, The University of Tokyo, Tokyo, Japan

**Keywords:** long-term oxygen therapy, positive airway pressure, home mechanical ventilation, national database

## Abstract

**Introduction:**

Japan is among the most rapidly aging countries worldwide, and the need for long-term respiratory care continues to grow. Long-term oxygen therapy (LTOT), home positive airway pressure (PAP), and home mechanical ventilation (HMV) are widely used for chronic respiratory diseases; however, nationwide long-term trends in their use remain insufficiently characterized. We aimed to examine recent national patterns in the use of these modalities.

**Methods:**

We conducted a descriptive study using National Database of Health Insurance Claims and Specific Health Checkups of Japan Open Data from fiscal years (FYs) 2017 to 2023. The annual claim counts for LTOT, PAP, and HMV were extracted. Population-adjusted rates (claims per 100,000 population) were calculated using national demographic data. Subgroup analyses were performed by sex across five age groups (0-24, 25-44, 45-64, 65-74, and ≥75 years), reflecting the clinically meaningful stages of aging in Japan.

**Results:**

The population-adjusted rate of LTOT use increased steadily across all years, age groups, and sexes. PAP use also increased consistently throughout the study period, with larger increases among older adults. In contrast, HMV use demonstrated only small overall changes, with a gradual increase that was most pronounced among adults aged 0-24 years, while a declining trend was observed among those aged ≥75 years. Similar patterns were observed in the age- and sex-stratified analyses.

**Conclusions:**

The nationwide use of LTOT and PAP increased between FY 2017 and FY 2023, whereas HMV use exhibited age-dependent variation. These findings indicate shifting needs in home respiratory support, with growing demand for LTOT and PAP and age-divergent patterns of ventilator use. Continued monitoring is essential to anticipate future service requirements and inform planning for age-appropriate home respiratory care.

## Introduction

Japan is among the most rapidly aging societies in the world, and this demographic transition has been accompanied by an increase in the number of individuals living with chronic respiratory diseases ^(1)^. Chronic obstructive pulmonary disease (COPD), interstitial lung disease, and sequelae of pulmonary tuberculosis have become leading indications for home respiratory care in Japan and constitute major public health challenges ^(1), (2), (3)^. In this context, the indications for long-term oxygen therapy (LTOT) have expanded to include conditions such as cluster headache in 2018 and long-term respiratory impairment following coronavirus disease 2019 (COVID-19) infection ^(4), (5)^. Positive airway pressure (PAP), including continuous PAP or adaptive servo ventilation, has become central to the management of sleep apnea syndrome (SAS) and chronic heart failure (CHF) and is typically administered during sleep ^(6)^. Home mechanical ventilation (HMV) is increasingly used for individuals with neuromuscular disorders, chest wall diseases, and advanced chronic respiratory failure ^(7)^. Additionally, national healthcare policies have promoted home-based medical care ^(8)^, a trend that was further reinforced during the COVID-19 pandemic ^(9)^. Together, these clinical and policy developments may have influenced national patterns in the use of home respiratory therapies.

Despite their growing clinical significance, nationwide data describing long-term patterns in the use of LTOT, PAP, and HMV remain limited. Most existing studies have focused on specific diseases or narrowly defined patient cohorts. To address this knowledge gap, we aimed to evaluate the nationwide temporal changes in claims for LTOT, PAP, and HMV using a national database.

## Materials and Methods

### Data source

This descriptive observational study used the National Database of Health Insurance Claims and Specific Health Checkups of Japan (NDB) Open Data, which are published annually by the Ministry of Health, Labor and Welfare. The NDB is one of the largest administrative claims databases worldwide and encompasses >95% of all health insurance claims under the Japanese universal healthcare system ^(10)^. The Open Data version provides aggregated and anonymized summaries derived from the full NDB. These summaries are stratified by sex, 5-year age categories, prefectural level, and service type, although they do not include information regarding clinical diagnoses, procedures, outcomes, comorbidities, or individual-level data.

### Study objective and variables

The primary objective was to describe temporal changes in the annual population-adjusted rates of LTOT, PAP, and HMV claims. In this study, LTOT, PAP, and HMV were defined according to the corresponding reimbursement management-fee codes in the NDB Open Data. These management-fee categories represent broad insurance-defined groups of home respiratory therapies rather than specific clinical modalities. The PAP category mainly reflects PAP therapies, such as continuous or bilevel PAP, which are typically administered during sleep for SAS, with a smaller proportion representing nocturnal PAP use for CHF. In contrast, the HMV category represents the use of home mechanical ventilators for chronic respiratory failure, including both invasive and noninvasive ventilation. However, the dataset does not distinguish between ventilator interfaces or specific clinical indications within the HMV category. Therefore, these classifications should be interpreted as reimbursement-based categories rather than strictly defined clinical modalities.

As supplemental information, we first examined the composition of HMV use by extracting two fee-addition categories related to HMV, including the fee addition for (1) positive-pressure ventilation delivered via tracheostomy and (2) noninvasive ventilator use. These add-on codes allowed us to descriptively assess trends in invasive and noninvasive ventilatory support within the broader HMV category. Additionally, we extracted annual claim counts for home nasal high-flow (NHF), which was newly reimbursed in 2022. NHF data were obtained in the same manner as those for LTOT, PAP, and HMV.

Annual claim counts for the management fees associated with these modalities were extracted for fiscal year (FY) 2017 through FY 2023. In Japan, each FY begins in April and ends the following March (e.g., FY 2017 refers to April 2017-March 2018). Management fees are reimbursement codes assigned to patients receiving home-based respiratory therapy under the national insurance system. In clinical practice, claims are submitted at intervals of 1-3 months depending on the follow-up schedule. Therefore, a single patient may generate multiple claims per year (approximately 4-12 claims annually). Because the NDB Open Data provide aggregated claim counts rather than patient-level data, these values reflect the volume of service utilization rather than the exact number of patients. Time-series plots were generated to visualize annual changes for each modality.

For descriptive purposes, we evaluated temporal patterns across the COVID-19 pandemic period. The study period was divided into three phases: prepandemic (FY 2017-2019), pandemic (FY 2020-2021), and postpandemic (FY 2022-2023). Population-adjusted rates were summarized for each period.

### Subgroup analyses

In addition to the overall analysis, subgroup analyses were performed by sex across five age groups (0-24, 25-44, 45-64, 65-74, and ≥75 years). These age categories, particularly those representing older adults, were selected for two reasons. First, demographic aging in Japan is rapid and uneven across age strata, and changes in the proportion of older adults can influence healthcare utilization trends ^(11)^. Second, major chronic respiratory diseases, including COPD, interstitial lung disease, and lung cancer, occur predominantly in older adults, with incidence and prevalence rising sharply after approximately 65 years of age ^(12), (13), (14)^. Furthermore, the threshold of 75 years is widely used in Japan to distinguish the “late elderly” population in public health statistics and the healthcare insurance system. Adults aged ≥75 years often demonstrate distinct patterns of multimorbidity, frailty, and care needs compared to those aged 65-74 years ^(15)^. Therefore, separating the two older age groups was considered necessary to capture changes specific to the oldest population segment and to better understand which demographic groups contributed most to the observed nationwide trends.

### Population adjustment

Demographic denominators for each FY were obtained from publicly available vital statistics issued by the Ministry of Health, Labor and Welfare ^(11)^. Detailed population data for each year are provided in Supplementary Table 1. Annual population-adjusted rates were calculated as the number of claims per 100,000 population. For stratified analyses, age- and sex-specific population denominators were applied to generate population-adjusted rates for each subgroup. Population adjustment was performed to account for demographic changes over time, particularly the expansion of the population aged ≥75 years, which could influence the interpretation of crude claim counts.

### Data suppression and statistical analysis

In accordance with the confidentiality policy of the NDB Open Data, small cell counts below 10 are masked. Masked values were handled as zero, following established practices in previous studies using this dataset ^(16)^. All analyses were performed using Stata/MP 18.0 (StataCorp LLC, College Station, TX, USA). As all information is anonymized and publicly available, institutional review board approval and informed consent were waived.

## Results

**Figure 1.**
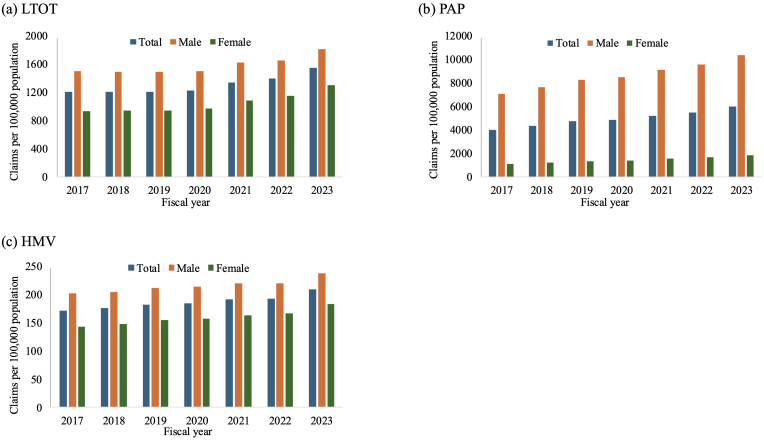
Annual nationwide trends in LTOT, PAP, and HMV use from FY 2017 to FY 2023. Population-adjusted annual claim rates (claims per 100,000 population) are shown for (a) LTOT, (b) PAP, and (c) HMV. All rates were adjusted using national population estimates for each FY. FY: fiscal year; HMV: home mechanical ventilation; LTOT: long-term oxygen therapy; PAP: positive airway pressure.

**Figure 2.**
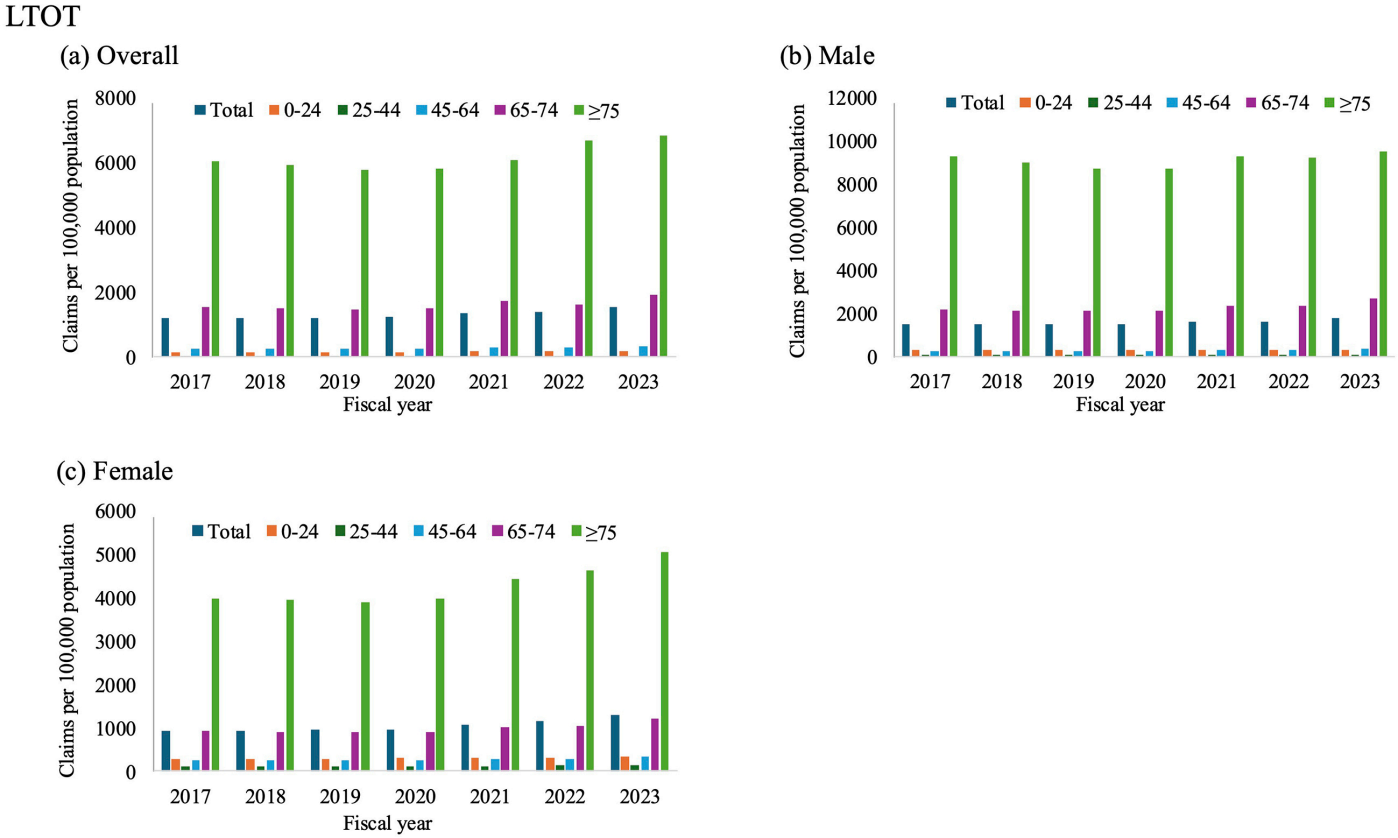
Annual trends in LTOT use by sex and age group from FY 2017 to FY 2023. Population-adjusted LTOT claim rates (claims per 100,000 population) are shown for the following groups: (a) overall, (b) male, and (c) female. Each panel includes age-stratified categories (0-24, 25-44, 45-64, 65-74, and ≥75 years). The rates were calculated using age- and sex-specific population denominators for each FY. FY: fiscal year; LTOT: long-term oxygen therapy.

From FY 2017 to FY 2023, the population-adjusted rate of LTOT claims increased steadily. The total annual rate rose from 1203.9 per 100,000 population in FY 2017 to 1545.2 in FY 2023 (Figure 1A). Age-stratified rates also increased over the same period, with values rising from 151.0 to 172.8 in the group aged 0-24 years, from 35.6 to 49.0 in the group aged 25-44 years, from 265.6 to 345.2 in the group aged 45-64 years, from 1538.7 to 1937.3 in the group aged 65-74 years, and from 6059.7 to 6833.6 in the group aged ≥75 years (Figure 2A). When stratified by sex, LTOT rates increased from 1493.4 to 1807.8 among males (Figure 2B) and from 929.5 to 1296.3 among females (Figure 2C).

**Figure 3.**
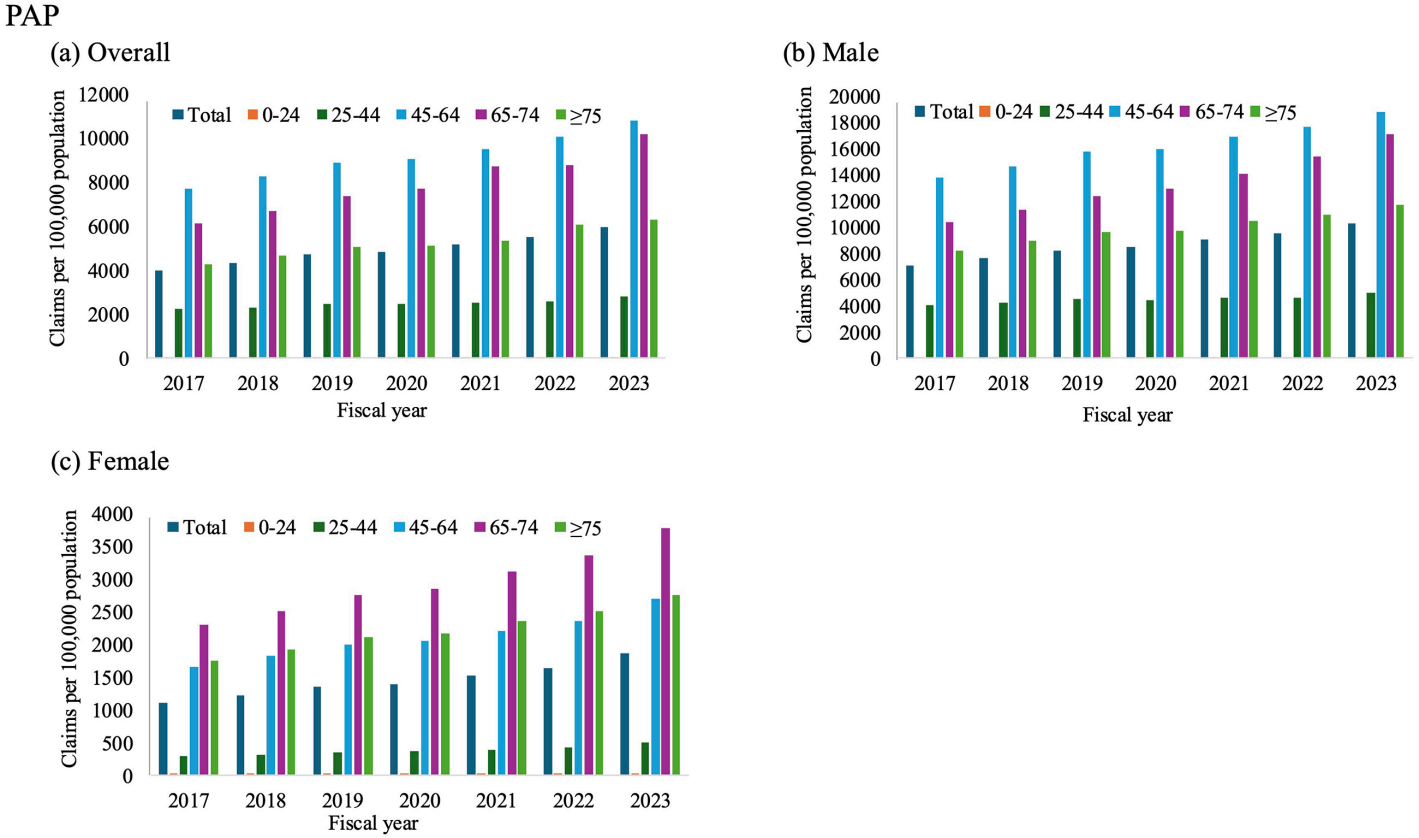
Annual trends in PAP use by sex and age group from FY 2017 to FY 2023. Population-adjusted PAP claim rates (claims per 100,000 population) are shown for the following groups: (a) overall, (b) male, and (c) female. Each panel includes age-stratified categories (0-24, 25-44, 45-64, 65-74, and ≥75 years). The rates were calculated using age- and sex-specific population denominators for each FY. FY: fiscal year; PAP: positive airway pressure.

For home PAP, the total population-adjusted rate increased from 4010.6 in FY 2017 to 5488.7 in FY 2021 and 5976.6 in FY 2023 (Figure 1B). Age-specific rates also increased, from 56.3 to 74.1 in the group aged 0-24 years, from 2230.2 to 2793.5 in the group aged 25-44 years, from 7708.8 to 10,794.1 in the group aged 45-64 years, from 6146.0 to 10,174.6 in the group aged 65-74 years, and from 4290.9 to 6311.9 in the group aged ≥75 years (Figure 3A). Sex-specific rates increased from 7076.1 to 10,329.1 among males (Figure 3B) and from 1105.1 to 1853.7 among females (Figure 3C).

**Figure 4.**
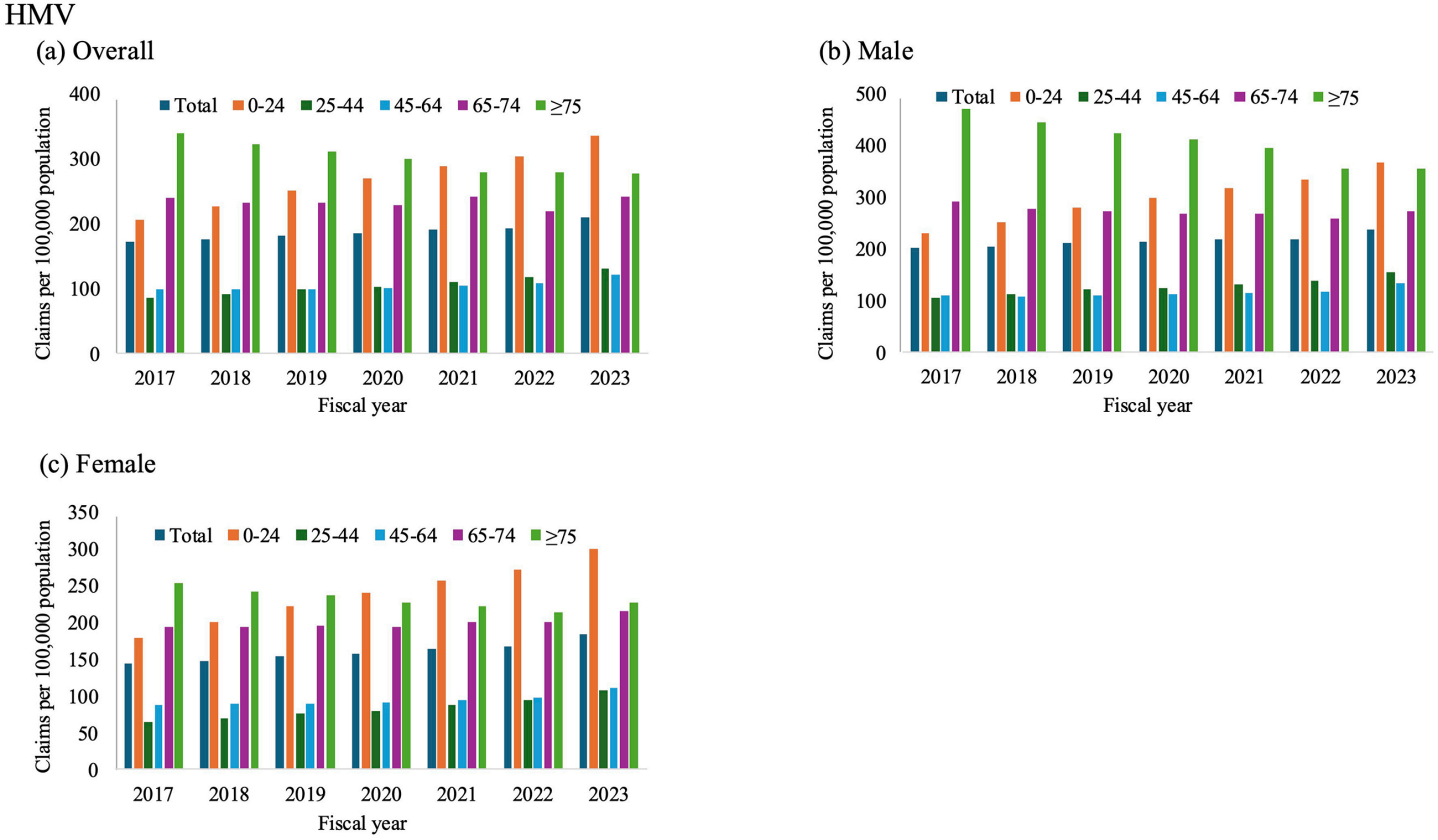
Annual trends in HMV use by sex and age group from FY 2017 to FY 2023. Population-adjusted HMV claim rates (claims per 100,000 population) are shown for the following groups: (a) overall, (b) male, and (c) female. Each panel includes age-stratified categories (0-24, 25-44, 45-64, 65-74, and ≥75 years). The rates were calculated using age- and sex-specific population denominators for each fiscal year. FY: fiscal year; HMV: home mechanical ventilation.

For HMV, the total population-adjusted rate increased more modestly than the corresponding rates for LTOT and PAP, rising from 171.3 in FY 2017 to 209.1 in FY 2023 (Figure 1C). In the age-stratified analysis, annual rates in four groups (0-24, 25-44, 45-64, and 65-74 years) either increased modestly or remained stable (e.g., 0-24 years: 204.9 to 334.4; 25-44 years: 84.9 to 131.2; 45-64 years: 98.5 to 122.3; and 65-74 years: 239.4 to 242.2) (Figure 4A). In contrast, the group aged ≥75 years demonstrated a gradual decline (from 338.6 in 2017 to 277.8 in 2023). In sex-stratified analyses, HMV rates increased modestly from 201.2 to 236.7 among males (Figure 4B) and from 143.0 to 183.0 among females (Figure 4C).

Overall, LTOT and PAP use increased consistently across all age and sex groups over time, whereas HMV use remained stable or increased modestly in younger and middle-aged groups and declined gradually in the group aged ≥75 years. A summary of the key findings, including total annual claims, population-adjusted rates, and percentage changes from FY 2017 to FY 2023, is presented in Table 1. When stratified by the pandemic period, the mean population-adjusted rates of LTOT and PAP progressively increased from the prepandemic to the pandemic and postpandemic periods. In contrast, HMV rates demonstrated only a modest increase across these periods. No clear disruptions associated with the COVID-19 pandemic were observed (Table 2).

**Table 1.** Comprehensive Summary of Home Respiratory Care Trends (FY 2017-FY 2023).

Modality	2017 claims	2023 claims	% change (claims)	2017 rate	2023 rate	% change (rate)
LTOT	1,525,380	1,921,506	+26.0%	1,203.9	1,545.2	+28.3%
PAP	5,081,693	7,432,041	+46.3%	4,010.6	5,976.6	+49.0%
HMV	145,985	163,636	+12.1%	171.3	209.1	+22.1%

Notes: Claims reflect service utilization volume and do not represent unique patient counts. Rates are expressed as claims per 100,000 population. HMV: home mechanical ventilation; LTOT: long-term oxygen therapy; PAP: positive airway pressure.

**Table 2.** Summary of Population-Adjusted Rates by Pandemic Period (FY 2017-FY 2023).

Modality	Prepandemic (FY 2017-FY 2019)	Pandemic (FY 2020-FY 2021)	Postpandemic (FY 2022-FY 2023)	% change (prepandemic–postpandemic)
LTOT	1205.6	1282.1	1469.3	+21.8%
PAP	4353.4	5016.1	5732.7	+31.7%
HMV	175.9	187.4	200.5	+14.0%

Values represent mean population-adjusted rates (claims per 100,000 population) for each period. HMV: home mechanical ventilation; LTOT: long-term oxygen therapy; PAP: positive airway pressure.

As supplemental findings, invasive ventilator use via tracheostomy increased gradually overall, with the clearest rise in the group aged 0-24 years (Supplementary Figure 1). Noninvasive ventilator use increased slightly but declined in the group aged ≥75 years (Supplementary Figure 2). NHF generated a small number of claims in FY 2022 and FY 2023 after its reimbursement introduction, with the highest use in the group aged ≥75 years (Supplementary Figure 3).

## Discussion

This nationwide analysis demonstrated that the use of LTOT and PAP increased steadily from FY 2017 to FY 2023 across all age and sex groups, whereas HMV use remained stable or increased modestly in younger adults and declined among those aged ≥75 years. These findings illustrate important shifts in home respiratory care in the context of Japan’s rapidly aging population.

Japan continues to experience a growing burden of chronic respiratory diseases, including COPD, interstitial lung disease, sleep disorders, and post-tuberculosis lung disease ^(3), (17), (18)^. As the number of older adults increases, the demand for long-term respiratory support is expected to rise, likely contributing to the continued expansion in LTOT and PAP use. Additionally, prolonged respiratory impairment following COVID-19 infection has been increasingly recognized ^(5)^, and the pandemic may have influenced the patterns of home oxygen use ^(9)^. However, our period-stratified analysis showed consistent increases across the prepandemic, pandemic, and postpandemic phases, suggesting that long-term demographic and clinical factors were the primary drivers of these trends. This may reflect the chronic and long-term nature of home respiratory care, which is less sensitive to short-term healthcare disruptions compared with acute care services.

In contrast, HMV use exhibited a different pattern, with a gradual decline in adults aged ≥75 years. A nationwide registry-based study from Sweden reported substantial increases in both the incidence and prevalence of HMV use over two decades, accompanied by changes in patient characteristics and a decline in the use of invasive ventilation ^(19)^. However, these findings reflect an increase in HMV use at the population level. In comparison, our results demonstrated age-dependent divergence, with declining use among older adults and modest increases among younger individuals. While invasive ventilation use decreased over time in the Swedish cohort, our analysis suggests a different pattern, with declining use among older adults (≥75 years) and increasing use among younger individuals. This difference may reflect variations in population structure, healthcare systems, and clinical practice patterns across the two countries. Several factors may have contributed to this observation. First, the increasing emphasis on advance care planning in Japan may lead to older adults declining long-term device-dependent therapies near the end of life, contributing to the decreasing use of home ventilators in this age group ^(20), (21)^.

Second, changes in the underlying disease profiles of patients receiving HMV may also have contributed to this trend. For example, the decline may partly reflect a reduction in patients with post-tuberculosis sequelae, as tuberculosis incidence has steadily decreased in Japan while treatment outcomes have improved, resulting in fewer older adults living with severe chronic respiratory impairment after tuberculosis ^(22)^.

Third, a system-level change occurred in April 2022 when home NHF became reimbursable under the national insurance program. Early utilization patterns provide contextual information regarding the introduction of this modality; however, its specific impact on HMV trends cannot be determined from the aggregated data. Home NHF may serve as an alternative to noninvasive ventilation for selected patients, potentially influencing clinical decisions ^(23), (24)^.

Finally, the COVID-19 pandemic may also have affected decisions regarding long-term invasive or noninvasive ventilation. Concerns regarding infection control, caregiver burden, and the feasibility of home management may have shaped patient and clinician choices during this period ^(25)^. The decline in HMV use among the oldest adults likely reflects a combination of evolving clinical practice, patient preferences, and system-level changes, whereas the observed increase in invasive ventilation use among younger individuals suggests a parallel expansion of long-term survival in chronic neuromuscular or structurally restrictive respiratory disorders. The marked increase among individuals aged 0-24 years may reflect the improved survival of patients with congenital or early-onset conditions, supported by advances in respiratory management and multidisciplinary care, as well as the growing number of patients transitioning from pediatric to adult care. These findings highlight a shift in the epidemiology of HMV, with an increasing contribution from younger patients with long-standing chronic conditions.

Concurrently, invasive ventilator use exhibited a modest overall increase, with the most pronounced growth observed among individuals aged 0-24 years. This pattern further supports the notion that the composition of the HMV population is shifting toward younger individuals who require prolonged ventilatory support ^(3), (26), (27)^. These age-dependent divergent trends indicate that the composition of the HMV population may be shifting toward younger individuals with chronic conditions requiring prolonged ventilatory support.

This study has several strengths. First, it uses nationwide data covering more than 95% of the Japanese population, summarizing real-world practice. Second, it enables comparisons across multiple home respiratory modalities within a unified framework. Third, the analysis captures age-specific trends, including divergent patterns of ventilator use. Fourth, the inclusion of the NHF following recent reimbursement changes provides timely insights into emerging therapies. Finally, the findings have direct relevance to healthcare planning in aging societies.

This study also has some limitations inherent to the use of administrative aggregated data. Management-fee claims do not directly correspond to the number of individual patients, as a single patient may generate multiple claims per year depending on follow-up intervals, and new and continuing users cannot be distinguished. Clinical information, including diagnoses, severity, and outcomes, is unavailable, and the influence of specific diseases or indications cannot be assessed. Additionally, COVID-19–related changes in home respiratory care cannot be isolated, and although we described early utilization of newly reimbursed home NHF, its specific impact on HMV or other modalities cannot be determined from these aggregated data. Finally, masked small counts may introduce minor underestimation, although the effect is likely limited. Despite these constraints, the NDB Open Data encompass nearly the entire insured population in Japan and offer a comprehensive view of nationwide utilization patterns of home respiratory therapies.

In this nationwide analysis, LTOT and PAP use increased steadily from FY 2017 to FY 2023, while HMV use demonstrated a contrasting pattern characterized by a decline among adults aged ≥75 years and a modest increase that was most evident among those aged 0-24 years. Rather than indicating a uniform shift in modality, these age-dependent differences suggest that the underlying drivers of home respiratory therapy use may vary across demographic groups. Factors such as changes in clinical practice, evolving patient preferences, the COVID-19 pandemic, and the introduction of home NHF may have contributed, although their individual effects cannot be separated in this dataset. Continued monitoring will be essential for planning home respiratory services and meeting future needs in aging societies. From a policy perspective, these findings have implications for workforce planning, resource allocation, and the development of home-care infrastructure in super-aging societies. The increasing demand for LTOT and PAP highlights the need for expanded home-based care capacity, whereas age-specific trends in HMV use suggest evolving patient preferences and care strategies.

## Article Information

### Author Contributions

Tomohiro Akaba contributed to study conception, data acquisition, data interpretation, and drafting of the manuscript. Hideki Katsura, Miki Kubo, and Etsuko Tagaya contributed to data analysis, data interpretation, and critical revision of the manuscript. All authors contributed to the drafting or critical revision of the manuscript, approved the final version, and agree to be accountable for all aspects of the work.

### Conflicts of Interest

None

### Institutional Review Board Information

As all information is anonymized and publicly available, institutional review board approval and informed consent were not required.

### Data Statement

The data used in this study were obtained from the NDB Open Database (https://www.mhlw.go.jp/stf/seisakunitsuite/bunya/0000177182.html) provided by the Ministry of Health, Labour and Welfare. All data used in this study were publicly accessible.

## Supplement

Supplementary Material

## References

[1] Muro S. Letter from the Japanese respiratory society. Respirology. 2025;31(1):102-4. 41243214 10.1002/resp.70160

[2] Yarbrough C, Miller M, Zulu M, et al. Post-tuberculosis lung disease: addressing the policy gap. PLoS Glob Public Health. 2024;4(9):e0003560. 39236033 10.1371/journal.pgph.0003560 PMC11376554

[3] Hamada S, Ueki J, Hirai T, et al. A summary of the Japanese White Paper on Home Respiratory Care 2024. Respir Investig. 2025;63(4):585-91. 10.1016/j.resinv.2025.04.019 40339468

[4] Cohen AS, Burns B, Goadsby PJ. High-flow oxygen for treatment of cluster headache: a randomized trial. JAMA. 2009;302(22):2451-7. 19996400 10.1001/jama.2009.1855

[5] Makam AN, Burnfield J, Prettyman E, et al. One-year recovery among survivors of prolonged severe COVID-19: a national multicenter cohort. Crit Care Med. 2024;52(7):e376-89. 38597793 10.1097/CCM.0000000000006258 PMC11176028

[6] Bradley TD, Logan AG, Lorenzi Filho G, et al. Adaptive servo-ventilation for sleep-disordered breathing in patients with heart failure with reduced ejection fraction (ADVENT-HF): a multicentre, multinational, parallel-group, open-label, phase 3 randomised controlled trial. Lancet Respir Med. 2024;12(2):153-66. 38142697 10.1016/S2213-2600(23)00374-0

[7] Akashiba T, Ishikawa Y, Ishihara H, et al. The Japanese Respiratory Society Noninvasive Positive Pressure Ventilation (NPPV) Guidelines (second revised edition). Respir Investig. 2017;55(2):83-92. 10.1016/j.resinv.2015.11.007 28012501

[8] Sun Y, Sakata N, Iwagami M, et al. Regional disparities in home health care utilization for older adults and their associated factors at the secondary medical area level: a nationwide study in Japan. Geriatr Gerontol Int. 2024;24(12):1350-61. 39522173 10.1111/ggi.15011 PMC11628894

[9] Shibata M, Aoki T, Matsushima M. Impact of the COVID-19 pandemic on home medical care utilization in Japan. J Gen Intern Med. 2024;39(14):3146-54. 39227543 10.1007/s11606-024-09003-2 PMC11618568

[10] Yasunaga H. Updated information on NDB. Ann Clin Epidemiol. 2024;6(3):73-6. 39034945 10.37737/ace.24011 PMC11254583

[11] Vital statistics of Japan. e-Stat. Ministry of Health, Labour and Welfare. 2025 [cited 2025 Dec 10]. Japanese. Available from: https://www.e-stat.go.jp/dbview?sid=0003411657

[12] Joshi PR. Pulmonary diseases in older patients. Geriatrics. 2024;9(1):34. 38525751 10.3390/geriatrics9020034 PMC10961796

[13] Kobayashi T, Nishino Y, Takiguchi T, et al. Epidemiological and therapeutic profiles of lung cancer patients in the Hokushin Region Japan: a retrospective hospital administrative database study. BMC Pulm Med. 2023;23(1):322. 37658334 10.1186/s12890-023-02610-5 PMC10472700

[14] Kotaki K, Ikeda H, Fukuda T, et al. Trends in COPD prevalence in elderly individuals in Japan. Int J Chron Obstruct Pulmon Dis. 2019;14(1):791-8. 31040658 10.2147/COPD.S189372 PMC6452819

[15] Ouchi Y, Rakugi H, Arai H, et al. Redefining the elderly as aged 75 years and older: proposal from the Joint Committee of Japan Gerontological Society and the Japan Geriatrics Society. Geriatrics Gerontology Int. 2017;17(7):1045-7. 10.1111/ggi.13118 28670849

[16] Akaba T, Arimura K, Kan-o K, et al. Geographic variation in bronchoscopy use across Japan. Respir Investig. 2025;63(5):1174-8. 10.1016/j.resinv.2025.09.017 40992123

[17] Tanabe N, Sato S. Narrative review of current COPD status in Japan. J Thorac Dis. 2021;13(6):3878-87. 34277077 10.21037/jtd-20-2263 PMC8264685

[18] Mirrakhimov AE, Sooronbaev T, Mirrakhimov EM. Prevalence of obstructive sleep apnea in Asian adults: a systematic review of the literature. BMC Pulm Med. 2013;13(1):10. 23433391 10.1186/1471-2466-13-10 PMC3585751

[19] Palm A, Grote L, Einarsson J, et al. Evolution of home mechanical ventilation in Sweden over 27 years. Chest Pulm. 2024;2(4):100108. 42548517 10.1016/j.chpulm.2024.100108 PMC13420463

[20] Inoue M, Hanari K, Hamano J, et al. Current engagement in advance care planning in Japan. Gerontol Geriatr Med. 2019;5(1):2333721419892694. 31903410 10.1177/2333721419892694 PMC6926984

[21] Kashiwagi M, Morioka N, Terajima M, et al. Awareness-raising activities of advance care planning for community residents: a nationwide cross-sectional survey in Japan. BMC Palliat Care. 2024;23(1):303. 39736597 10.1186/s12904-024-01635-9 PMC11686830

[22] Annual report of tuberculosis registration and surveillance in Japan 2024 [Internet]. Ministry of Health, Labour and Welfare, Japan. 2024 [cited 2025 Dec 10]. Japanese. Available from: https://www.mhlw.go.jp/stf/seisakunitsuite/bunya/0000175095_00016.html

[23] Jácome C, Jácome M, Correia S, et al. ‘Effectiveness, adherence and safety of home high flow nasal cannula in chronic respiratory disease and respiratory insufficiency: a systematic review. Arch Bronconeumol. 2024;60(8):490-502. 38782632 10.1016/j.arbres.2024.05.001

[24] Nagata K, Horie T, Chohnabayashi N, et al. Effectiveness, adherence and safety of home high flow nasal cannula in chronic respiratory disease and respiratory insufficiency: a systematic review. Am J Respir Crit Care Med. 2022;206(11):1326-35. 35771533 10.1164/rccm.202201-0199OC PMC9746854

[25] Shah AJ, Suh E-S, Kaltsakas G, et al. COVID-19 impact on home mechanical ventilation services. Respir Med. 2022;197(1):106831. 35366623 10.1016/j.rmed.2022.106831 PMC8958773

[26] Saito T, Kawai M, Kimura E, et al. Effectiveness, adherence and safety of home high flow nasal cannula in chronic respiratory disease and respiratory insufficiency: a systematic review. Neuromuscul Disord. 2017;27(2):107-14.28003112

[27] Tagami M, Kimura F, Nakajima H, et al. Tracheostomy and invasive ventilation in Japanese ALS patients. J Neurol Sci. 2014;344(1-2):158-64. 25017882 10.1016/j.jns.2014.06.047

