# Supplementary material for: Diverging Trends in Home Respiratory Care in an Aging Society: National Data from Japan
Source: JMA J. 2026 Jun 12;9(4):966–73. doi: 10.31662/jmaj.2025-0583 (PMC13453931; doi:10.31662/jmaj.2025-0583)

Supplementary material

Supplementary table 1. Annual population (total and by sex) as of October 1

Fiscal year	population	Overall	0-24	25-44	45-64	65-74	Over >75
2017	Overall	126,706	27,815	30,730	33,009	17,670	17,481
2017	Male	61,655	14,268	15,618	16,508	8,427	6,835
2017	Female	65,051	13,547	15,111	16,502	9,243	10,648
2018	Overall	126,443	27,651	29,946	33,268	17,602	17,975
2018	Male	61,532	14,189	15,236	16,651	8,400	7,058
2018	Female	64,911	13,463	14,711	16,619	9,202	10,919
2019	Overall	126,167	27,418	29,261	33,603	17,395	18,490
2019	Male	61,411	14,074	14,908	16,831	8,312	7,288
2019	Female	64,756	13,343	14,354	16,773	9,083	11,202
2020	Overall	126,146	27,057	29,072	33,988	17,425	18,602
2020	Male	61,350	13,863	14,815	17,032	8,336	7,305
2020	Female	64,797	13,196	14,258	16,958	9,089	11,297
2021	Overall	125,502	26,627	28,462	34,199	17,541	18,673
2021	Male	61,019	13,642	14,508	17,147	8,389	7,333
2021	Female	64,483	12,986	13,954	17,051	9,151	11,342
2022	Overall	124,947	26,278	28,016	34,417	16,872	19,365
2022	Male	60,758	13,475	14,291	17,268	8,070	7,653
2022	Female	64,189	12,803	13,726	17,150	8,802	11,708
2023	Overall	124,352	25,903	27,671	34,551	16,149	20,077
2023	Male	60,492	13,290	14,137	17,350	7,730	7,985
2023	Female	63,859	12,613	13,533	17,201	8,419	12,093

All population counts are shown after dividing the raw values by 1,000 (unit: 1,000 persons).

Supplementary figure legend

Supplementary Figure 1. Annual population-adjusted rates of invasive ventilator use (fee addition for positive-pressure ventilation via tracheostomy) from FY 2017 to FY 2023 by (a) overall, (b) male, and (c) female.

This category reflects patients receiving invasive home mechanical ventilation through a tracheostomy. Rates were calculated using age- and sex-specific population denominators for each fiscal year.

Supplementary Figure 2. Annual population-adjusted rates of noninvasive ventilator use (fee addition for home mechanical ventilation without tracheostomy) from FY 2017 to FY 2023 by (a) overall, (b) male, and (c) female.

This category reflects home use of noninvasive ventilators for chronic respiratory failure. Rates were calculated using age- and sex-specific population denominators for each fiscal year.

Supplementary Figure 3. Annual population-adjusted rates of home nasal high-flow (NHF) therapy from FY 2017 to FY 2023 by (a) overall, (b) male, and (c) female.

NHF became reimbursable under the national insurance program in FY 2022; therefore, values from FY 2017 to FY 2021 are shown as zero, indicating that no reimbursable home

NHF claims existed during these years. Rates were calculated using age- and sex-specific population denominators for each fiscal year.

Supplementary Figure 1

HMV(invasive ventilator)

(a) Overall

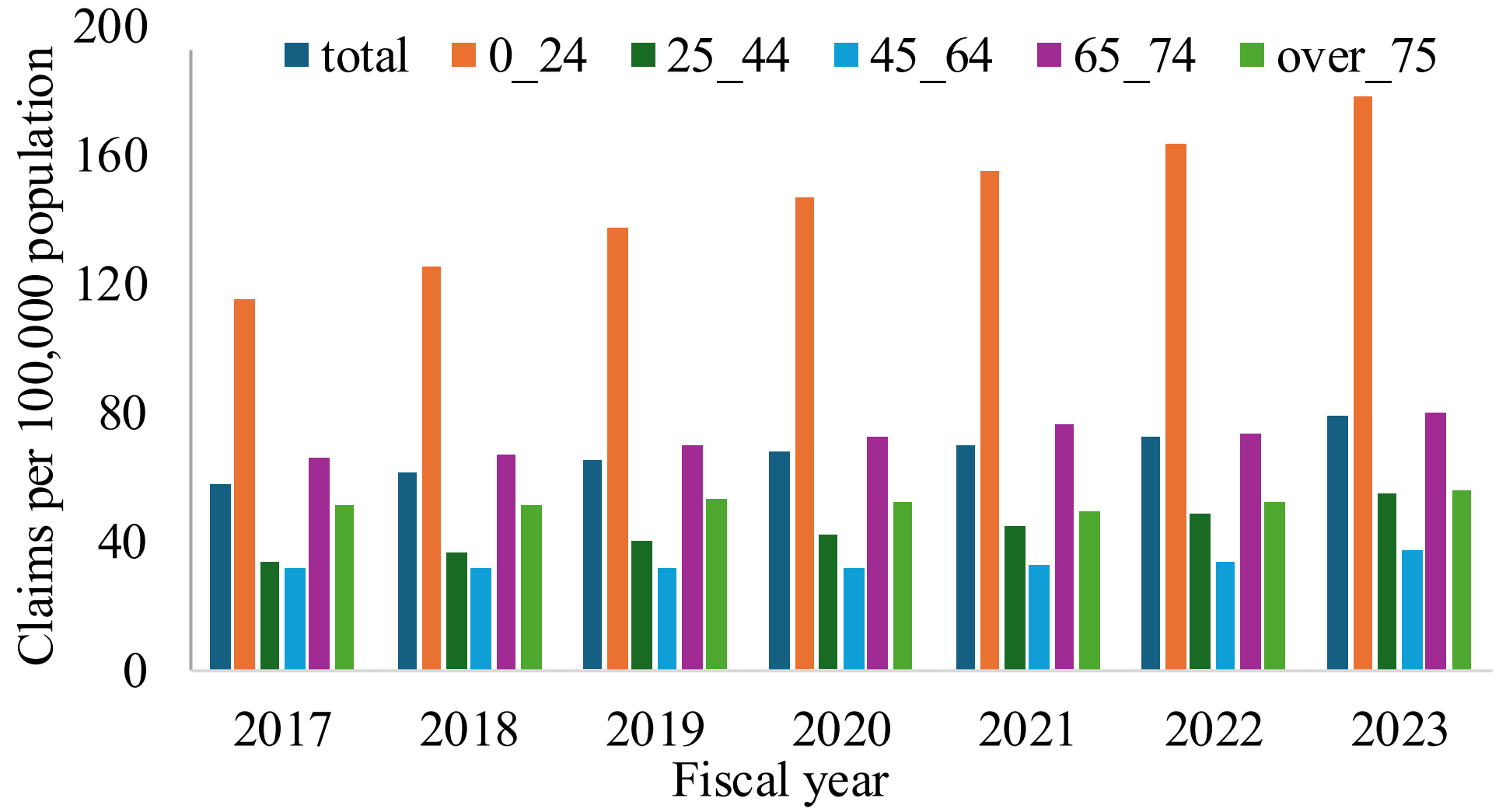

(b) Male

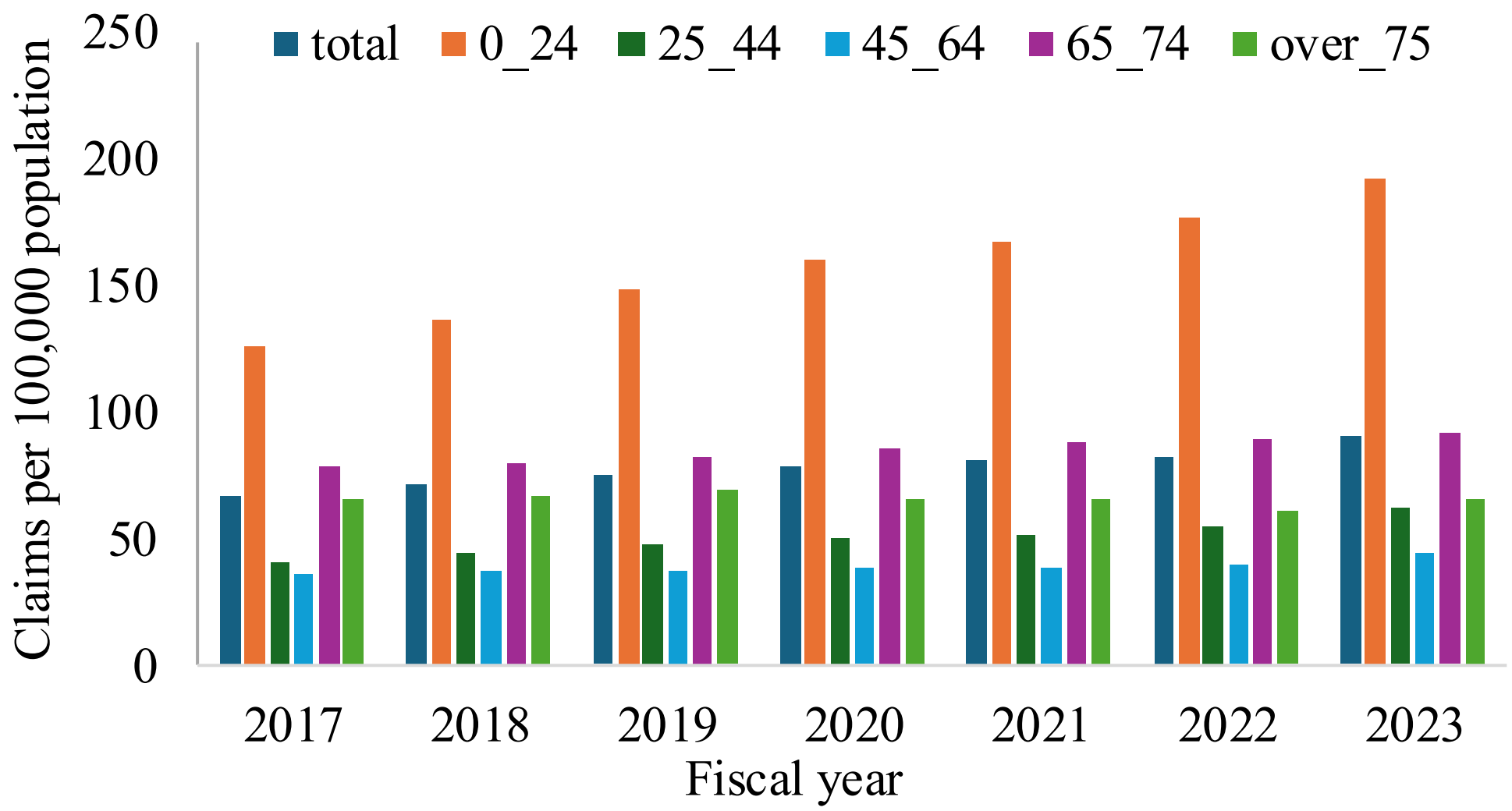

(c) Female

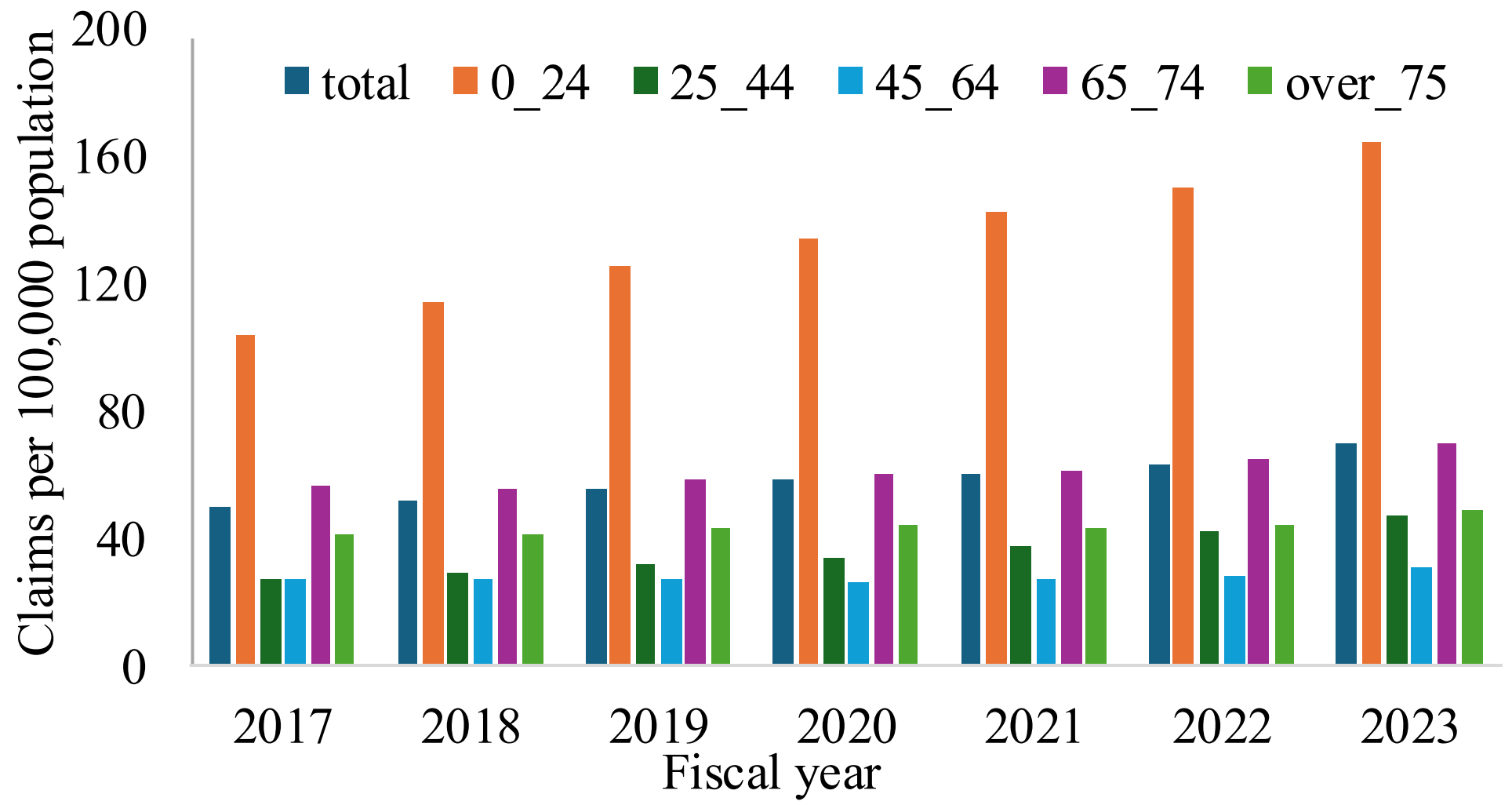

Supplementary Figure 2

HMV(noninvasive ventilator)

(a) Overall

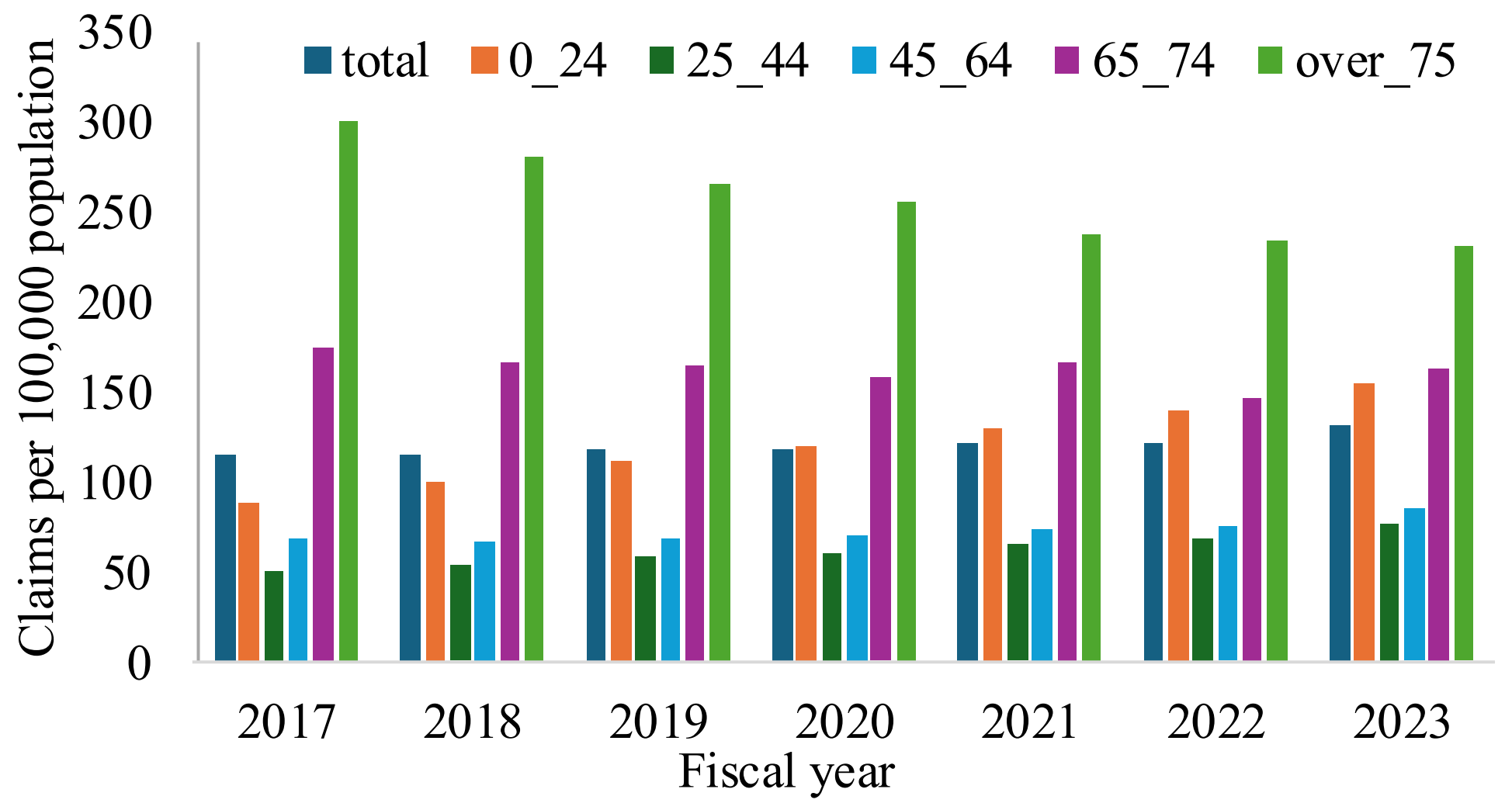

(b) Male

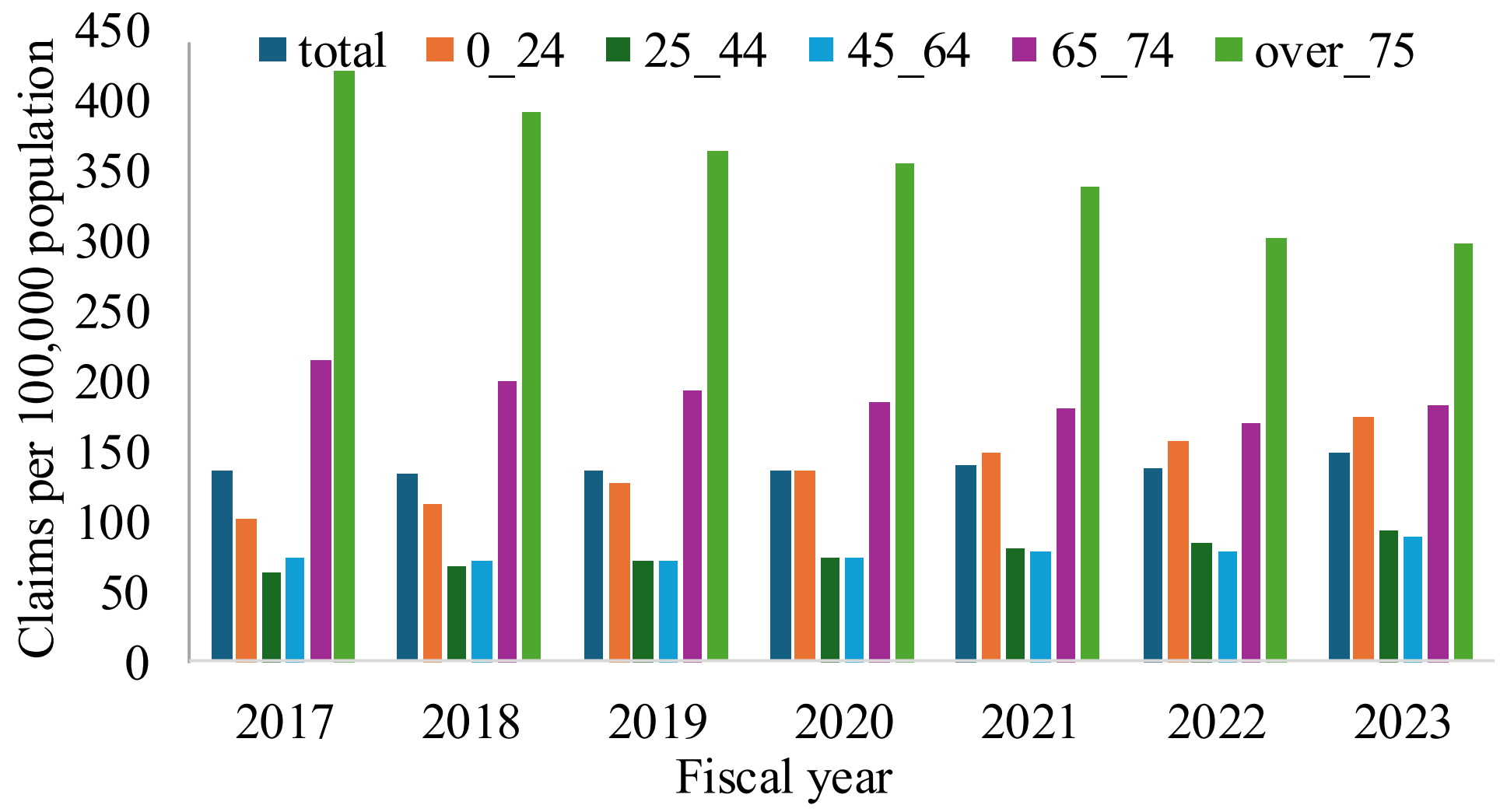

(c) Female

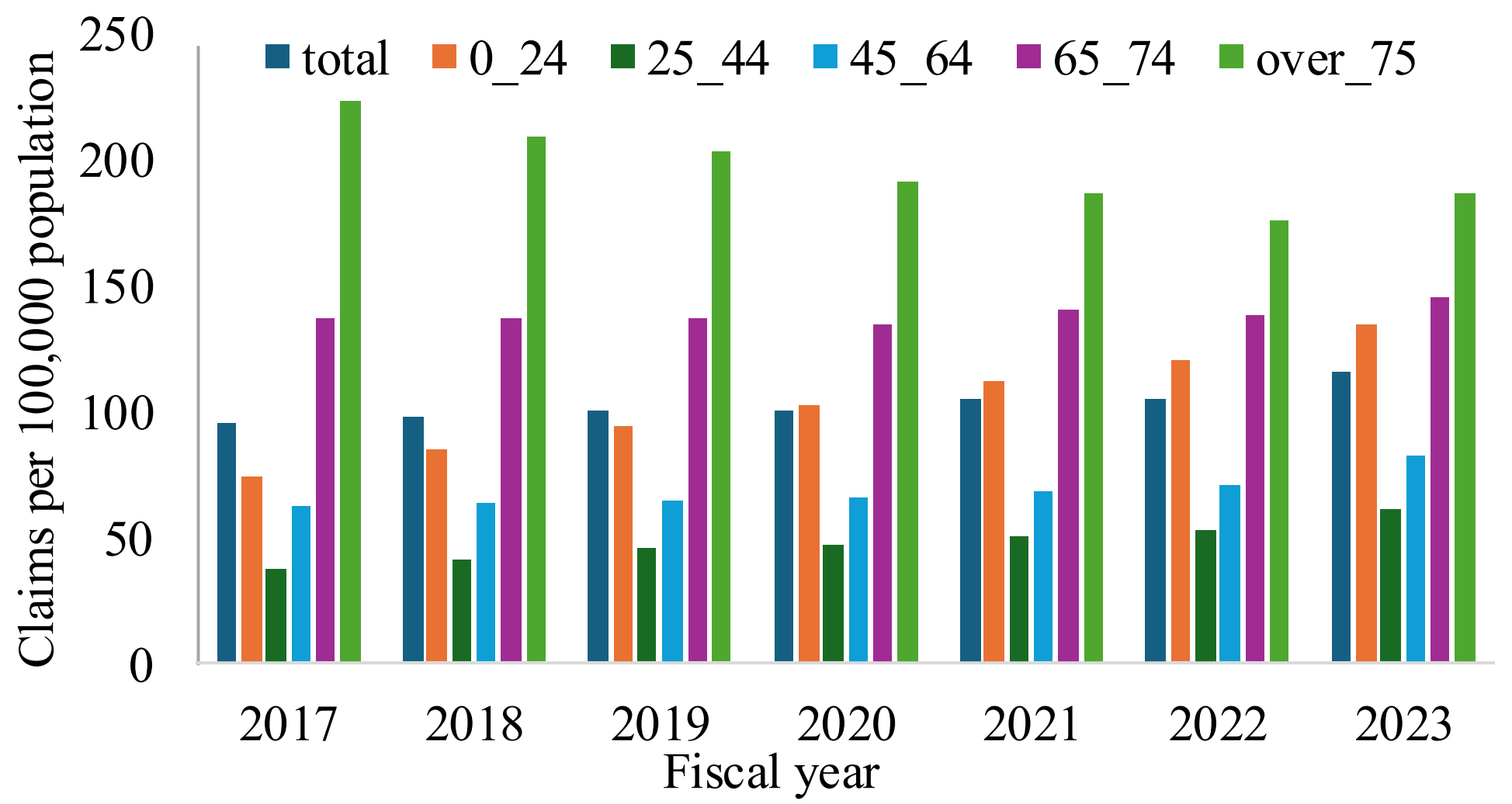

Supplementary Figure 3

NHF

(a) Overall

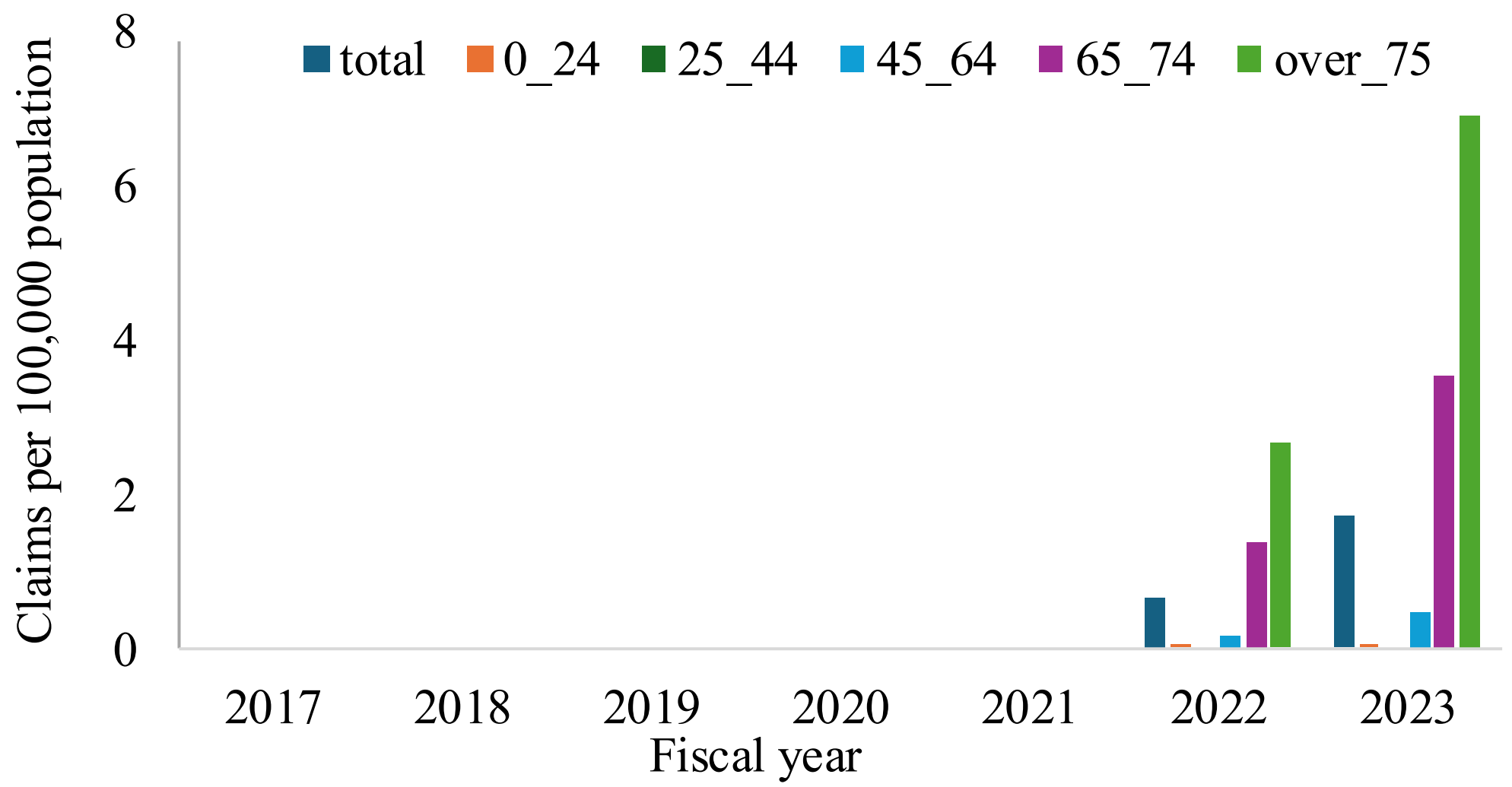

(b) Male

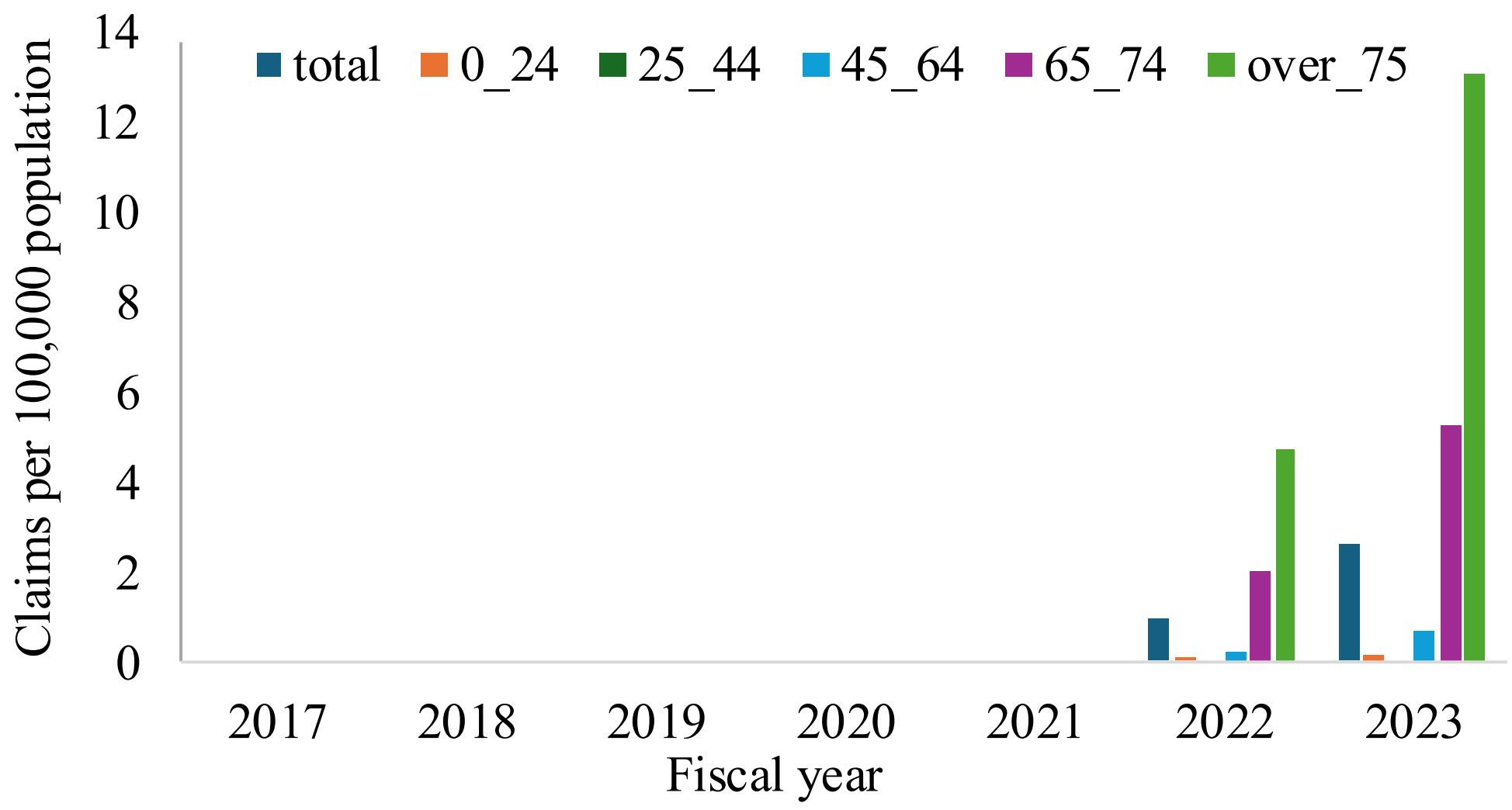

(c) Female

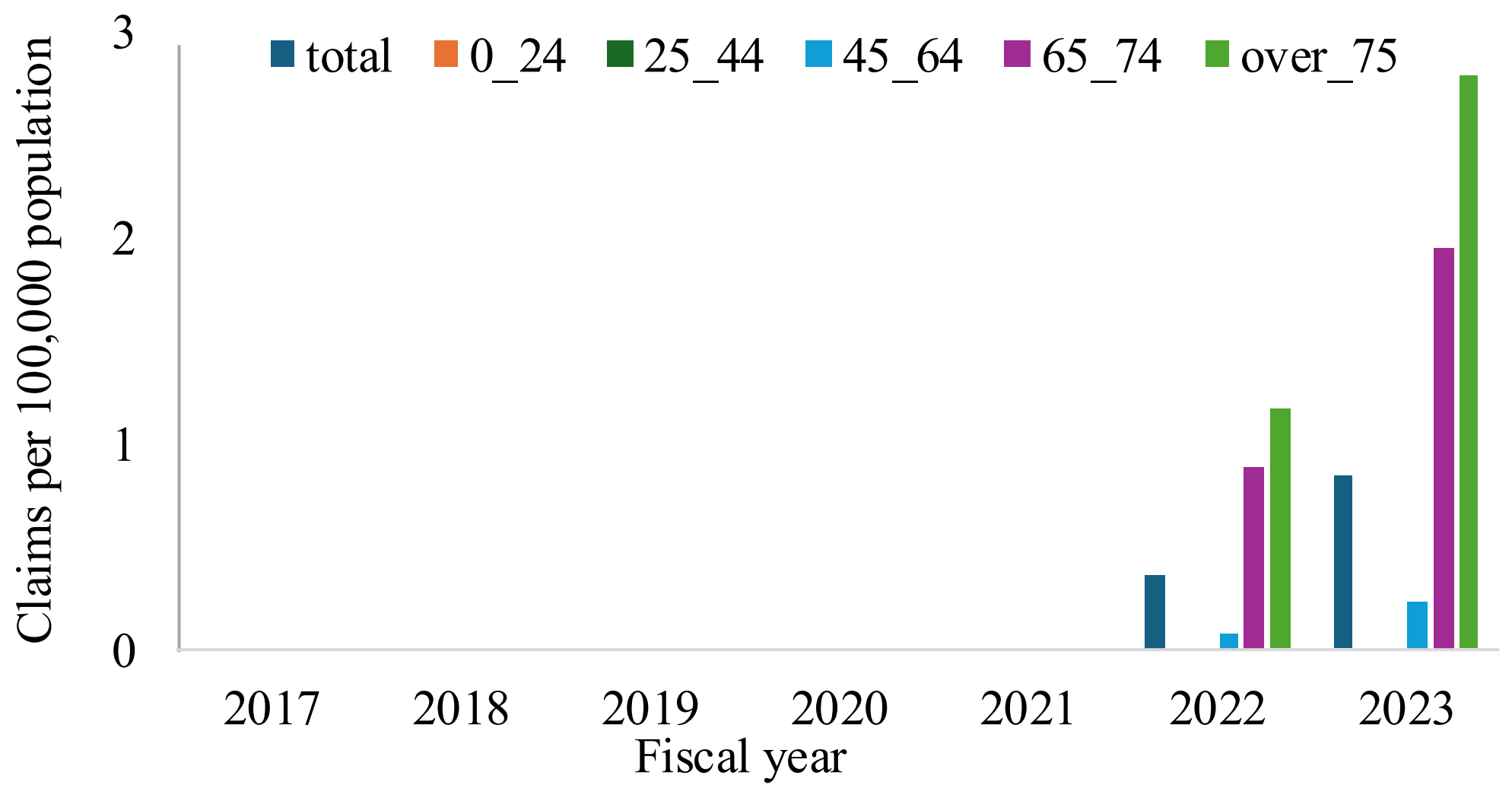

Supplement: Supplementary file 1 [file 2433-3298-9-4-0966-s001.pdf]
